# Supplementary material for: Validation of the mental health continuum-short form: The bifactor model of emotional, social, and psychological well-being
Source: PLoS One. 2022 May 18;17(5):e0268232. doi: 10.1371/journal.pone.0268232 (PMC9116639; doi:10.1371/journal.pone.0268232)
Supplement: Attachment — Submitted filename: Response to Reviewers.docx [file pone.0268232.s001.docx]

Dear Reviewers,

Thank you for taking your time to review and comment on the paper titled,” Validation of the mental health continuum-short form: The bifactor model of emotional, social, and psychological well-being”

The followings are the changes that we have made to the paper in accordance with your feedback:

We have acknowledged Reviewer #1’s feedback regarding the lengthy introduction and background information given. We have significantly reduced the background information and have limited the introduction of each core concept to only one paragraph. However, we have also recognised the importance of highlighting the shortcomings of other measures, the adequate description, and prior psychometric evaluations of the current measure, as per commented by Reviewer #2, hence we have kept majority of the latter part of the introduction. We hope that by doing so, we provide readers a succinct yet comprehensive literature review of the matter.

Reviewer’s #2 recommendation of including more specific references to previously validated wellbeing scales within local context was not able to be addressed because we followed Reviewer #1’s recommendations to focus on the scale and it’s psychometrics. With regard to highlighting the local need of the scale, we hope by extensively highlighting the flaws of existing measures and having noted the lack of validation studies of the tool in local context, we can justify the need for the current investigation into the measure.

In addition, we have also recognised the concern raised by Reviewer #2 regarding the sampling method, and thus, have added more comments within the limitations section to highlight the potential generalisation issues.

The data of the study have been made public and can be found on figshare’s depository. https://doi.org/10.6084/m9.figshare.19174205.v2.

Lastly, we have made changes to the manuscript so it now meets PLOS ONE’s style requirements, as requested by the Academic Editor.

Once again, thank you for your time reviewing and providing valuable comments on the paper. I hope we have adequately addressed the concerns that each of you have raised.

Best Regards,
Zack Yeo Zhishen
Lidia Suarez
