# Supplementary material for: Validation of the mental health continuum-short form: The bifactor model of emotional, social, and psychological well-being
Source: PLoS One. 2022 May 18;17(5):e0268232. doi: 10.1371/journal.pone.0268232 (PMC9116639; doi:10.1371/journal.pone.0268232)
Supplement: Attachment — Submitted filename: Response to Reviewers.docx [file pone.0268232.s002.docx]

Dear Reviewers,

Thank you for taking your time to review and comment on the paper titled,” Validation of the mental health continuum-short form: The bifactor model of emotional, social, and psychological well-being”

The followings are the changes that we have made to the paper in accordance with your feedback:

We have acknowledged your feedback of completing the well-being measures review section of the paper by including the recent well-being instrument developed and published by Bonacchi et al. (2021). We have added a new paragraph towards the end of the section (line 147-152) to acknowledge the instrument, including its pros and limitations.

Once again, thank you for your time reviewing and providing valuable comments on the paper. I hope we have adequately addressed the feedback.

Best Regards,
Zack Yeo Zhishen
Lidia Suarez
